# Personalization in Australian K-12 classrooms: how might digital teaching and learning tools produce intangible consequences for teachers’ workplace conditions?

**DOI:** 10.1007/s13384-022-00530-7

**Published:** 2022-04-28

**Authors:** Janine Aldous Arantes

**Affiliations:** Victoria University, Footscray, Australia

**Keywords:** Personalisation, Teachers, Human rights, Algorithmic bias, Data

## Abstract

Recent negotiations of ‘data’ in schools place focus on student assessment and NAPLAN. However, with the rise in artificial intelligence (AI) underpinning educational technology, there is a need to shift focus towards the value of teachers’ digital data. By doing so, the broader debate surrounding the implications of these technologies and rights within the classroom as a workplace becomes more apparent to practitioners and educational researchers. Drawing on the Australian Human Rights Commission’s *Human Rights and Technology final report*, this conceptual paper focusses on teachers’ rights alongside emerging technologies that use or provide predictive analytics or artificial intelligence, also called ‘personalisation’. The lens of Postdigital positionality guides the discussion. Three potential consequences are presented as provocations: (1) What might happen if emerging technology uses teachers’ digital data that represent current societal inequality? (2) What might happen if insights provided by such technology are inaccurate, insufficient, or unrepresentative of our teachers? (3) What might happen if the design of the AI system itself is discriminatory? This conceptual paper argues for increased discourse about technologies that use or provide predictive analytics complemented by considering potential consequences associated with algorithmic bias.

## Introduction

The use of commercial apps and platforms in K-12 educational settings across Australia has dramatically grown in recent years. Many schools across Australia are now either ‘Google schools’ or ‘Microsoft Schools’ (Perrotta et al., 2021), which are in addition to multitudes of other commercial apps and platforms used as digital teaching tools (Arantes, 2020; Rennie et al., 2019). This network of people, companies, applications and devices in the classroom (Kumar et al., 2019) is supported by a dominant educational discourse that largely places technology as an innovation (Gonski et al., 2018). For example, benefits include analysing data about learners and their contexts to understand better and optimise learning and the environments in which it occurs (Siemens, 2013). Undoubtedly, positive implications of such analytics in education are apparent (Shibani et al., 2020). Likewise, there are growing concerns (Perrotta et al., 2021).

Conversely, despite less widely researched, the skills required by teachers to negotiate such tools place pressure on already stretched schools to prepare them to work in these new employment contexts (Mosely et al., 2021; Selwyn, 2019a). This pressure presents a problem in that teachers’ innovative use of digital teaching tools is considered largely in terms of pedagogy and educational practice, with consequences for labour less well researched. This paper focusses on the digital data produced when teachers use digital teaching tools and their rights as a human in the workplace and is significant as the data which flow from teachers’ use of digital teaching tools potentially have consequences for their human rights.

This paper presents a nuanced perspective to the debate about digital teaching tools that use or offer personalisation via predictive analytics in the classroom. The paper does not focus on technology that enables personalisation in terms of pedagogy and educational practice. Instead, this paper considers how emergent technology such as personalisation may impact teachers’ human rights based on a report produced by the Australian Human Rights Commissioner Edward Santow (AHRC, 2021). This paper has a focus on the predictive insights that underpin personalisation. Personalisation is used here to describe the data-driven outputs often termed ‘insights’ from predictive analytics that compare individual datasets to benchmarked averages. Of most interest in this paper is personalisation to aid teachers’ personalised learning and personalisation for commercial purposes of providing business intelligence to firms providing digital tools to schools. It conceptualises how the Australian Human Rights Commission’s reports about the impact of technology, offers ways to address the various intangible consequences of technology’s use in education.

Consequences considered in this paper focus on the computational process of algorithmic bias. Algorithmic bias is described here as “predictions or outputs from an AI system, where those predictions or outputs exhibit erroneous or unjustified differential treatment between two groups” (Lattimore et al., 2020, p. 21). With personalisation, a form of business intelligence in educational settings, this paper also considers personalisation in terms of commercialisation (Arantes, 2020). This conceptual paper argues for increased discourse about technologies that use or provide predictive analytics by considering potential strategies to address consequences associated with algorithmic bias. A brief note on terminology follows, followed by a discussion of the theoretical positioning that underpins the discussion and how it helps understand the changing face of digital data in educational settings.

### A note on terminology

Terminology remains conflated when working in interdisciplinary research such as this. For example, terms such as automated decision making, personalisation and artificial intelligence (AI) are broad terms that lack definitive definition across fields. Terminology has implications for this paper. For example, some may argue that automation represents repetitive tasks based on programmed commands or algorithms, whereas AI is for non-repetitive tasks. Others may use the term AI to describe predictive analytics. For the purpose of this conceptual paper, the focus is on the forms of technology that provide predictive insights by comparing an individual dataset to benchmarked averages in larger datasets and utilises the term personalisation to do so.

In educational settings, personalisation provides insights about student learning and targeted advertising, talent analytics and increasingly claims to predict staff mental health and welfare. For example, previous research terminology has included automated analytical software (Zuboff, 2019) or technology that can ‘learn’ insights from the abstraction and combination of digital data across multiple contexts (Perrotta et al., 2021). Further, the digital data used to formulate insights have been beneficial to teachers when used and re-used to increase time efficiencies and convenience (Seimens, 2013) and surveil and control (Zuboff, 2019). The argument presented in this paper does not attempt to define terms or comment on innovation. Rather, it focusses on the exposure of teachers to technology and their use of it, the technology that uses and provides insights from teachers’ digital data and the proxies of their data used to formulate insights. Conceptually, a Postdigital lens, specifically Postdigital positionality (Hayes, 2021), has been utilised to provide a personal approach to studying Postdigital contexts. Personalisation and the teachers’ data that enable it are considered in relation to their individual human rights to explore how digital teaching and learning tools might produce intangible consequences for teachers’ workplace conditions. This theoretical underpinning and its relationship to digital data used in personalisation are described in the following section of the paper.

## Theoretical positioning and the changing face of ‘data’ in education

Postdigital theory adopts a way of thinking that the digital is no longer novel, and digital innovation is now mundane. Postdigital theory provides a means of looking beyond specific apps or platforms used pedagogically by teachers. It examines the digital, non-digital, commercial and datafied material that informs and modulates social relations that exist in educational systems (Fawns, 2019). Postdigital theory encourages consideration of broader contexts. For example, in this instance, consider how the Australian Human Rights Commission addresses intangible consequences of digital data and emergent technologies in various workplaces. It encourages us to look at educational data and technological innovations differently.

At the surface, a lot of data in K-12 education could be perceived as being neither big nor obfuscated and black boxed (Pasquale, 2015) in data infra-structures. Instead, data are primarily considered student assessments, NAPLAN data, or something explored through Excel, being stored and used locally (Selwyn, 2020). The postdigital encourages us to consider commercial digital data in educational settings and its consequences for teachers’ rights. Whether at work or working at home, teachers’ data are collected and used by commercial apps and platforms as part of their fundamental means to complete their job. Business practices in education are widely discussed as the ‘ed-tech market’ (Williamson, 2021).

Further, the sales and marketing strategies use teachers as conduits for educational technology advertising within the relatively unregulated ‘ad-tech market’ (Andrejevic et al., 2021). It is the data associated with concepts described as Servitization (Arantes, 2020), Assetization (Komljenovic, 2020) and Platformization (Kumar et al., 2019). By considering this data as valued and important, the notion of data shifts from the perspective of student learning (Siemens, 2013) and local data (Selwyn, 2020) and towards teacher labour, agency and privacy (Selwyn, 2019b; Williamson, 2017). Further, by focussing on the teachers’ workplace as a space embedded with personalisation tools, we are prompted to discuss human rights issues associated with algorithmic bias (Barocas & Selbst, 2016; Birhane & van Dijk, 2020; Crawford & Schultz, 2014). However, how such data are collectively used and its implications for individual teachers remains a fundamentally under-considered component of what constitutes data in educational settings. Whilst student data and NAPLAN data are important, teachers’ de-identified commercially collected, collated and used data in ed-tech and ad-tech markets is a form of data yet to be given the same priority in day-to-day life discussions.

Teachers’ data have commercial value, which arguably obfuscates the consequences of rights-based concerns. Teachers’ digital data have value because it is being used in automated decision making and the technological process of personalisation. Whilst I do not focus on the commercialisation of educational systems in-depth, the perspective presented here has emerged from my consideration of commercial digital data in educational systems and its potential impact on human rights. For example, Perrotta and Selwyn (2019) state, “Indeed, the past few years have witnessed the rise of general-purpose predictive infra-structures with large technology companies” (p. 15). Secondly, Gulson and Sellar (2019) argue that such infrastructures are changing governance structures in educational systems. Thirdly, the Australian Human Rights Commission (AHRC, 2021) discusses a need for responsible innovation. Suppose we consider the Australian Human Rights Commission’s argument regarding the commercial digital data in education as a commodity and capacity to govern. In that case, teachers should be able to take advantage of the promise of new technologies whilst still upholding their human rights.

Specifically, I consider personalisation tools according to the postdigital theory of postdigital positionalities (Hayes et al., 2021). Postdigital positionality is helpful in this context, as it indicates that datafication “plays out differently in each individual human context” (Hayes et al., 2021, p. 73). Through this lens, I consider how modern educational systems are shaped via data infrastructures (Jandrić, 2020) and can affect individual teachers (Hayes et al., 2021). In attempting to explain ‘how’ we may address these consequences, I argue that through this lens, we see that we need to begin with a strategy to protect teachers’ workplaces through policy whilst at the same time promoting antitrust measures. That is, to start by not trusting claims made about personalisation, automated decision making and innovative AI. This argument is built on work demonstrating the utility of postdigital theory in its analysis of education (Fawns, 2019; Knox, 2019) and the synthesis of human values in terms of algorithmically informed platforms (Williamson, 2017). It adds to the discussion about postdigital teacher identities and clarifies teachers’ consequences alongside the changing face of data due to personalisation (Arantes, 2021).

### Postdigital positionality and the changing face of teachers’ personal data

Postdigital positionality provides a subjective approach to the study of postdigital contexts. For example, Arantes (2021) discusses the concept of Postdigital Teacher Identities, described as “a teacher’s identity actualization that works through algorithmic systems which infer categories of identity(s) on de-identified or anonymized data being positioned within established policy and guidelines” (p. 452). These identities are created from different forms of teachers’ data without explicit awareness for use by external stakeholders through proxies to represent the teachers. Proxies are used to provide insights and recommendations (O’Reilly, 2007). Proxies are data assemblages representing the teachers in automated decision making and the predictive insights used in personalisation. Although only a correlation, proxies have been widely used to drive behavioural change (Yeung, 2016) and predict diverse areas of people’s public and private lives (Kosinski et al., 2016). Based on de-identified data from past events, such predictions are often designed to optimise and personalise the everyday experience (Pessach & Shmueli, 2020). For example, Google Classroom provides automated attendance reports and automated assessments (Perrotta et al., 2021) to personalise learning. The process of personalisation uses these digital identities or proxies to compare to benchmark averages and provide insights and recommendations to personalise experiences. Postdigital positionality encourages us to consider the notion of *personal data* in automated decision making and personalisation.

Personal data are identifiable and protected under privacy legislation. There are limited personal data specifically about the individual teacher for a commercial organisation to use in practice. Although, Pangrazio and Selwyn (2020) state, “‘Big social data’…are an important component of decision making in fields ranging from financial credit through to job recruitment” (p. 1). Where personal data are authenticated, such as employee numbers and government emails (Norberg et al., 2007), big social data are de-identified as ‘Big Data’ focussing on social phenomena. To use personal data to make predictions would be using data for purposes other than where it was collected and could identify an individual (Clarke, 2008; Solove, 2006). To do so would violate various aspects of privacy legislation (Culnane & Leins, 2020). Thus, raising a significant point about commercial big data in education. Valid insights and recommendations are difficult to make decisions about without personal data (Ntoutsi et al., 2020). Even though copious amounts of big data are available from teachers’ trialling and using educational technology as part of their working conditions, intermediate proxies must be used to underpin data-driven decisions, insights and recommendations. That is, insights are fundamentally derived from de-identified metadata about a teacher (Williamson, 2021) or risk privacy breaches. On the flip side, the individual can be re-identified (Culnane & Leins, 2020; Culnane et al., 2017) with increasing amounts of metadata.

Considering the Australian Human Rights Commission call for responsible innovation in relation to Postdigital positionality, notions such as discrimination and unfair profiling in automated decision making and personalisation can be unpacked in educational settings. These topics have been explored using a Postdigital lens. For example, Hayes et al. (2021) posit the notion of Postdigital assemblages to have an impact on educational systems in terms of disadvantage and inclusion, and Hurley and Al-Ali (2021) refer specifically to the gendered effects of emergent technologies from a Postdigital perspective. By drawing on this theoretical framing, we are encouraged to acknowledge that no singular human or company profits from teachers’ use of technology alone. That is, new modes of governance have been established (Gulson & Sellar, 2019; Perrotta & Selwyn, 2019) due to the changing face of data, and there is a need to unpack associated impact in terms of fluid and datafied forms of discrimination explored in real-world contexts.

What follows is a description of the Australian Human Rights Commission’s *Human Rights and Technology final report* (referred to as ‘*The Report*’). *The Report* is used to reposition our understanding of ‘data’ alongside emergent ways in which data are being used through personalisation. The paper then discusses the changing face of data and the emergent technological process of personalisation concerning algorithmic bias. Personalisation has been chosen as a technology to unpack due to the Gonski report’s interest in personalised learning. Following this, three questions based on scenarios presented in *The Report* are considered, and potential consequences detailed in *The Report* are aligned to educational contexts. By referring to the findings of *The Report*, the next section of the paper unpacks the practical implications for teachers’ rights as humans who consume educational technology in their workplace through a Postdigital lens.

### The Human Rights and Technology final report: *‘The Report’*

The Australian Human Rights Commission’s *Human Rights and Technology final report* (referred to as ‘*The Report*’ moving forward) was chosen to scaffold how we might garner a deeper understanding of educational systems regarding human rights and technological implications. Further, ‘*The Report*’ was also selected as it simulates decision-making processes reflective of standard business practices that use or provide AI decision making in educational settings in relation to human rights. That is a subjective approach to studying a postdigital context in education. Educational environments are widely accepted to be commercialised (Hogan et al., 2018), and as such, business practices as part of educational settings are now commonplace. Due to the rapid growth of new and emerging commercial technologies in education, the decision-making processes that *The Report* highlights as areas needing attention is a pressing and urgent consideration for educational policymakers.

*The Report* communicates the Commission’s findings of 3 years of research and collaboration with large Australian organisations such as the CSIRO, CHOICE, the Consumer Policy Research Centre and Gradient Institute (AHRC, 2021). The collaborative approach provided a series of recommendations for both law and other reforms, ultimately adding to the ‘Digital Economy Strategy’ (previously the Digital Australia Strategy). Aiming to provide a similar opportunity for educational researchers, policymakers and administrators. By drawing on the Australian Human Rights Commission’s *Human Rights and Technology final report* (AHRC, 2021), this paper adds a perspective to the discourse about technology in educational contexts across Australia. It provides a valuable systematisation of critical themes that require attention from a perspective of teacher labour.

*The Report* is divided into four parts, (A) A national strategy on new and emerging technologies, (B) Artificial intelligence in decision making, (C) Regulation and an AI Safety Commissioner and (D) Accessible technology for people with disabilities (AHRC, 2021). This paper focusses on *The Report*ing of Part (B), Artificial intelligence in decision making, particularly the discourse surrounding ‘Algorithmic Bias’. AI in decision making is of interest; as Hayes et al. (2021) posit, these groups or postdigital assemblages constructed to make decisions from data impact educational systems in terms of disadvantage and inclusion. The section in *The Report* based on algorithmic bias was chosen as it is a fundamental process associated with technology that provides insights and recommendations to offer personalisation.

*The Report* provides three scenarios that focus on algorithmic bias. Each scenario is based on standard business practices impacted by algorithmic bias and explores potential real-world impacts in the financial services, telecommunications, energy and human resources sectors (AHRC, 2021). These three scenarios are connected to the context of education to respond to the question ‘How might digital teaching and learning tools produce intangible consequences for teachers’ workplace conditions?’ In considering the findings of the Australian Human Rights Commissioner Edward Santow’s 3-year project into the human rights implications of new technologies such as artificial intelligence (AHRC, 2021), I draw on the project’s findings but in the context of education. Through the use of provocations, I explore tensions discussed below.

This paper positions the digital as no longer new; digital innovation as mundane and explores teachers’ workplaces in terms of commercial big data and human rights guidelines. This broader context is significant as the same legislation presented in *The Report*, applies to teachers’ data and associated impacts to them as humans, with human rights. It deliberately shifts focus from technology and educational practice, pedagogy and learning, towards promoting understandings of educational issues through consideration of broader publications as there is a need for “greater guidance for government and non-government bodies in complying with anti-discrimination law in the context of AI-informed decision making (Recommendation 18)” (AHRC, 2021, p. 195). As such, this paper partly responds to this recommendation and attempts to inject this nuanced perspective into educational discourse about technology that uses digital data to provide insights. A response to the three provocations follows a description of algorithmic bias in educational settings.

## Algorithmic bias in educational settings

Algorithmic bias is foundational within predictive modelling and personalisation, unable to be removed from AI systems (Green & Chen, 2019; Green & Viljoen, 2020). It is apparent in all recommendations and insights with varying impacts, mainly as a result of (1) using data that represent current societal inequality, (2) the use of inaccurate, insufficient or unrepresentative data and (3) the design of the AI system itself (AHRC, 2021). In simple terms, algorithmic bias refers to the validity of insights and recommendations derived from the teachers’ aggregated de-identified and personalised data. Algorithmic bias is a fundamental component of personalisation and the predictive analytics that drive it. To explain, inherent in the process of personalisation is a tradeoff between accuracy and bias (Ntoutsi et al., 2020); thus, algorithmic bias is a fundamental aspect of the algorithmic systems explored in this paper (Friedler et al., 2016). Algorithmic bias, also called algorithmic fairness, is largely under-negotiated by teachers (Arantes, 2019), resulting in erroneous predictions informing educational practice and data-driven decision making. Note—‘data-driven decision making’ refers to commercial data, not NAPLAN or assessments in Excel, and as such, a decision could be employment or promotion-based insights and recommendations. Erroneous predictions can result in discrimination as these numerical proxies can also correlate to protected factors such as gender, race and ethnicity. Friedler et al. (2016) argue that the removal of algorithmic bias cannot be guaranteed and as such algorithmic bias is implicit within personalisation.

The implications of using automated decision making in education are discussed in terms of algorithmic bias (Baker & Hawn, 2021; Lattimore et al., 2020). Considering such implications is significant, as the practical implications of algorithmic bias in educational settings, which are teachers’ places of employment, have not yet received sufficient debate (Baker & Hawn, 2021; Perrotta & Selwyn, 2019). These implications are considered a topic of interest to teachers and schools who mandate their use and the educational technology providers themselves.

The consequences of algorithmic personalisation are intangible and, thus, problematic, but how these consequences are intangible relies on educators to understand automated decision making and other complex computational topics. For example, *The Report* states, “Algorithmic bias can sometimes have the effect of obscuring and entrenching unfairness or even unlawful discrimination in decision making” (AHRC, 2021, p. 13). Thus, if the Australian Human Rights Commission is aware that unlawful discrimination in decision making may occur, yet do not have the means to explain these consequences, strategies to address implications associated with algorithmic bias in educational settings should not be burdened on teachers and schools.

It is understood that data outputs built from commercial data in educational settings may result in erroneous insights and recommendations, which arguably would detract from profits (ACCC, 2019; Adamson et al., 2012). It is also understood that the surfeit of research concerning discriminatory practices results from predictive analytics in fields outside of education. From policing to the judiciary (Lightbourne, 2017), in talent analytic tools, platforms that claim to predict mental health and wellbeing, recruitment and promotion tools, as well as targeted advertising on the teachers’ social media and the ed-tech advertisements they are exposed to (Faliagka et al., 2012; McGuire & Ladd, 2014) algorithmic bias is problematic. Thus, the dominant discourse about technology as an ‘innovation’ needs to shift towards antitrust measures. This call aligns with talk about AI and, as such algorithmic bias in education “in controversial and circumspect terms, rather than accepted as a computational *fait accompli*” (Perrotta & Selwyn, 2019, p. 267).

In exploring *The Report*, associated consequences are studied as part of the discourse surrounding predictive modelling in consumer contexts. Educational technology businesses are increasingly collecting teachers’ *personal* data, which can improve AI systems, including how they assess teachers as potential customers. Their assessment arguably aims to increase profitability more so than educational outcomes. *The Report* explains how a commercial organisation may pursue maximum profitability. Acknowledging that this is not unlawful; it is the situations whereby it is unlawful to rely on *automated decision making that produces biased results* where a Human Rights focus lies. Stating that

This is certainly true of an AI system that produces discriminatory results. Put simply, a business that makes decisions using an AI system that exhibits algorithmic bias faces several legal, financial and reputational risks that need to be carefully and conscientiously addressed. (AHRC, 2021, p. 16)

There is no denying that educational outcomes must be met for technology to be considered; however, a commercial platform and app are first and foremost commercial. As such, teachers arguably “have little choice about whether they are subjected to these almost-ubiquitous data-collection practices” (AHRC, 2021, p. 16). I argue that the commercial platform must prove how they address issues of algorithmic bias before being deployed into a school setting. A contract of terms whereby the commercial organisation bears the burden of explanation before profit can be optimised. The burden of justification is justified, as *The Report* demonstrates that this is a ‘society at large’ issue. This is a tension that is felt within and beyond educational settings.

This tension has been explored in terms of how digital teaching and learning tools produce intangible consequences for teachers’ workplace conditions; the following provocations have been used. They situate the tension within the context of Australian educational settings. The paper will draw on three scenarios that align with the three causes of algorithmic bias discussed in The Report to understand and explore these provocations. The first provocation considers data representing society inequality through a scenario involving educational technology platforms marketing to teachers. It asks, ‘What might happen if emerging technology uses teachers’ digital data that represents current societal inequality?’ The second provocation considers a scenario where there is unrepresentative data and stereotyping of women in support positions and asks, ‘What might happen if insights provided by such technology are inaccurate, insufficient, or unrepresentative of our teachers?’ The third provocation considers the algorithms’ design that provides insights and recommendations in terms of talent analytics and asks, ‘What might happen if the design of the AI system itself is discriminatory?’ These provocations are explored through a Postdigital lens to unpack how intangible consequences are tangible.

### What might happen if emerging technology uses digital data to represent current societal inequality?

This paper aligns the AHRC scenario to educational technology platforms assessing the ‘value’ of advertising to specific teachers. These platforms may plausibly use automated decision making through data acquired via a data broker to evaluate teachers as prospective consumers. *The Report* details a simulation of a retail electricity market and associated AI systems that target consumers for contracted offerings.

The scenario begins by contextualising that targeted advertising is based on the assessment of whether a school or teacher as a potential customer is likely to be profitable. *The Report* identifies that profitability is dependent on income. Those schools with an income below average will likely result in lower profitability. This assessment may include the likelihood that the teacher or school may financially engage with the cost of the commercial platform, which may also include engaging with teachers who may influence other teachers (Dousay et al., 2018). For example, a new educational technology platform may enact a business strategy that offers ‘freemium’ apps. The app is provided for free or via subscription to secure market share (Arantes, 2020). Suppose an educational technology platform assesses that a potential teacher or school is likely to encounter problems paying for their product. In that case, the commercial platform may have little financial incentive to offer their product. Although the free platform can benefit the teacher or school, they may settle for a poorer product if the paid platform is more beneficial to the teacher or school.

Acknowledging that “consumers may not be given adequate information to make an informed choice about whether an offer is in their best interests” (AHRC, 2021, p. 19), *The Report* flags concerns about “situations where individuals may not be offered a market-competitive contract due to bias or discrimination” (p. 19). As *The Report* details, known attributes associated with socioeconomics are postcode, which aligns with protected attributes such as race and ethnicity. In the context of education, this may be where lower socio-economic schools, in comparison to wealthy private schools, are not offered equivalent educational resources from commercial platforms. Postcode may, however, also align with historical and current disadvantages (Barocas & Selbst, 2016; Beer, 2017).

Drawing on postdigital theory, we can look at postcode data differently and explore whether it may represent social inequity in the digital teaching and learning tools used in educational settings. Specifically, how modern educational systems are shaped via data infrastructures, and the postcode acts as a proxy with intangible consequences for teachers’ workplace conditions. Through this lens, notions such as discrimination and unfair profiling of teachers in automated decision making and personalisation can be considered. By doing so, we can consider implications associated with teachers who are Aboriginal and Torres Strait Islanders. *The Report* identifies the presence of “historical and current disadvantage experienced by Aboriginal and Torres Strait Islanders, compared with other people in Australia” (p. 33), which may be perpetuated in automated decision-making processes. Is it possible that the data may result in automated decisions associating “Aboriginal and Torres Strait Islander [teachers] with having a below-average income, and therefore identify them as likely to be less profitable” (AHRC, 2021, p. 33)? Regardless of whether a particular Aboriginal or Torres Strait Islander teacher or school with students who are Aboriginal or Torres Strait Islander is or is not profitable to the commercial platform; such a decision may contravene the Racial Discrimination Act 1975 (Cth) (Racial Discrimination Act) or even, corresponding state and/or territory law (AHRC, 2021). Broader discussion about teachers and schools receiving disparate offerings (Barocas & Selbst, 2016), although intangible due to the nature of the targeted advertising used by the commercial platform (Pasquale, 2015), is required.

### What might happen if insights provided by such technology are inaccurate, insufficient, or unrepresentative of our teachers?

*The Report* discusses a scenario whereby historical bias arose when data used to train an automated decision system no longer accurately reflects reality due to inaccurate, insufficient, or unrepresentative data (AHRC, 2021). The scenario provided by AHRC (2021) refers to women who historically have faced barriers to leadership roles and employment opportunities due to caring responsibilities. In this scenario, “13% fewer women [were] predicted by the AI system as profitable” (AHRC, 2021, p. 26), mainly because the training data do not identify a reduced gap in women and men’s income. Thus, although structural inequality reduced over time, an increased gap remained in the training data used to provide insights and recommendations.

The data do not reflect current reality, leading to unequal opportunity. For example, if a platform were used to predictively filter an applicant for a leadership role in education using inaccurate or insufficient data based on men generally holding leadership roles, the predictive models and resultant insights and recommendations would not be valid. This scenario in terms of educational settings has received research. For example, Anderson et al. (2019) examined factors impacting graduation, noting that of their sample, only 44 learners were of American Indian heritage. As such, the data were under representative of American Indians, rendering the insights invalid. Further, Baker (2019) also states what many educational researchers are acutely aware of: it is much harder to collect data in some schools than in others. Under representative data present a bias that can be produced into insights and recommendations, perpetuating inaccurate findings, which again remains ‘black boxed’ for those interpreting the data.

A second concrete example is when the Houston district board used commercial algorithmic systems in the United States to make transparent and hold to account teachers’ impact by analysing test scores over time. However, the results were then used to dismiss teachers deemed ineffective by the system without the algorithmic decision-making process being explained (Dawson et al., 2019). As the algorithmic systems were proprietary and not able to be scrutinised due to their commercial nature, they were considered “a potential violation of the teachers’ civil rights” (Dawson et al., 2019, p. 34). It is clear that commercial platforms and the algorithmic systems that underpin them interconnect educational settings with a broader infrastructure, only recently being scrutinised according to teachers’ rights.

*The Report* discusses ways in which mitigation of such discrepancies has been approached. A common approach is to remove protected attributes from the dataset. The goal is that the automated decision would no longer take those protected attributes into account. However, this strategy has proven problematic as proxies for protected attributes, as discussed above with postcode and socioeconomics, remain.

Drawing on postdigital theory within the context of K-12 educational settings, we can explore how inaccurate insights that inform digital teaching and learning tools may produce intangible consequences for teachers’ workplace conditions. Hurley and Al-Ali (2021) refer to gendered impacts of emergent technologies from a Postdigital perspective. By drawing on this theoretical framing, I argue that gender bias is perpetuated in the products that are used in educational settings. This argument is supported in research that provides tangible examples of gendered technologies. For example, Gutierrez (2021) notes that audio data, speech recognition systems and voice user interfaces have a pervasive gender bias. Google’s speech recognition systems to Apple’s Siri and Amazon’s Alexa are automated speakers that use female voices in ‘support roles’. The AHRC (2021) scenario demonstrates that automated decision making can still disadvantage women even when mitigation strategies are employed. Suppose, within the context of a school setting, a similar decision was made. In that case, this “could contravene the Sex Discrimination Act 1984 (Cth) (Sex Discrimination Act), or corresponding state or territory law.” (AHRC, 2021, p. 34). The binary selection choice of many automated educational products is trending towards forms of indirect discrimination. This tension must be included in consideration of the value of AI-informed technologies in and around educational settings.

### What might happen if the design of the AI system itself is discriminatory?

In taking steps to reduce algorithmic bias, *The Report* notes that removing protected attributes has been equated with avoiding direct discrimination. This design feature encourages the design of the AI itself to be of perceived importance. However, as discussed above, it may perpetuate forms of indirect discrimination. It is not necessarily unlawful to make decisions by reference to sex. *The Report* refers to the labels that influence the mathematical model produced as insights and recommendations in this third and final scenario. In this scenario, unconscious or conscious bias is manifest in the design of how insights and recommendations are produced.

The scenario produced by the AHRC (2021) refers to Southeast Asian Australians engaging with a call centre and their interactions forming the dataset used for automated decisions. Southeast Asian Australians were used in this scenario due to increased reports of racist treatment throughout the COVID-19 pandemic. The scenario describes how automated decisions depicted this cohort in society as less profitable. In terms of educational contexts, this aligns with the use of talent analytics and the employment of teachers. Used by employers to garner insights about how to recruit best, promote and justify termination of employees, e-recruitment and talent acquisition (Dutta, 2018) use predictive analytics to link “various data streams using appropriately defined unique identifiers, to get a complete picture of [teacher] behaviour” (Baesens et al., 2016, p. 813). By doing so, employers can forecast cultural fit, employee mental health, behaviour and performance (He et al., 2015), although arguably invalid due to algorithmic bias. As educational researchers, we need to push the boundaries of research beyond technology being used as an innovative tool and storing data locally and only on Excel. We need to explore how algorithmic interventions and their consequences in context (Green & Viljoen, 2020) and how they may impact teachers. A discriminatory AI system will produce intangible and arguably tangible consequences for teachers’ workplace conditions.

## Discussion

The key tensions presented in this paper need to be more widely researched and discussed in terms of teachers in their workplace and the associated impacts and implications. The dominant educational discourse in Australian educational settings largely places technology, particularly artificial intelligence, as innovation, and this must be complemented with a rights-based discourse. The skills required by teachers to negotiate such tools, whilst informed by how such tools may impact their rights, are severely lacking. Although there is a growing pressure on schools to prepare teachers to work in these new employment contexts, with artificial intelligence collecting and curating their data in and around the classroom, little pressure is applied to the platforms themselves. Antitrust measures must be implemented when digital teaching and learning tools shift local data to commercialised big data. Further, educational policy needs strategies enacted to address how big data, proxies and their capacity to circumnavigate current privacy legislation and impact teachers’ human rights.

Commercial platforms are mining educational data for profit and new technological developments (Perrotta & Selwyn, 2019). They are establishing emergent forms of data brokering (Williamson, 2020) and are turning them into profitable and distinctively global data streams for revenue. Whether it be location data, engagement data, device data, or the time a teacher logged onto their device, each piece of commercial data can be de-identified. When used as a proxy, it can infer new information according to commercial drivers for profit. As a proxy is distinctly different from the individual teacher’s personal data, the Australian Consumer and Competition Commission is calling for a change to the definition of personal information in current privacy legislation. The call seeks to change the definition of personal information in the Privacy Act “to clarify that it captures technical data such as IP addresses, device identifiers, location data and any other online identifiers that may be used to identify an individual” (ACCC, 2019, p. 456). Proxies are aggregated data collected within and around multiple classrooms (Arantes, 2021). They are widely used to benchmark averages and create ‘like’ teacher profiles or proxies, which are then used to underpin data-driven decisions (Kitchin, 2014). Thus, the dominant discourse surrounding the collection and use of teachers’ data and the validity of technologies that use or provide predictive analytics must begin by (1) acknowledging that commercial platforms do not deal in Excel spreadsheets and store data locally and (2) there are consequences for teachers’ rights.

The consequences are twofold. Firstly, seemingly objective predictions using big data have been shown to perpetuate social inequality in the form of data-driven insights and recommendations (Beer, 2017; Crawford & Paglen, 2019). Secondly, personal data or data that can be causally linked back to the user has been shown to violate privacy legislation (Culnane & Leins, 2020; Culnane et al., 2017). As society remains unaware of such connections due to the relative intangibility of how their data are connected and used (Beer, 2017; Pasquale, 2015), there is a need for strategies to actualise these concepts. This paper has done so by contextualising *The Report* (AHRC, 2021) in the context of teachers’ workplaces. The purpose of actualising the discourse was to illuminate teachers’ rights regarding the potential consequences of personalisation and algorithmic bias. By doing so, this paper has positioned the digital as no longer new and digital innovation as mundane whilst calling for policy development to protect teachers’ workplaces from commercial big data flow according to human rights legislation. Although also calling for antitrust measures, the main argument in this paper as a result of this discussion is to hold stakeholders who benefit from teachers’ cumulative big data whilst circumnavigating various rights, to account.

*The Report* recommends “greater guidance for government and non-government bodies in complying with anti-discrimination law in the context of AI-informed decision making (Recommendation 18)” (AHRC, 2021, p. 195) and complimenting the discourse with a rights-based approach will encourage this new perspective to be considered about the validity of technology in specific contexts. This paper has unpacked *The Report* to develop a deeper understanding of the human rights implications associated with new and emerging technologies in Australia’s context of educational settings. It unpacked the notion of data as a distinctly ‘non-local’ factor with commercial value in proxies being used for various commercial purposes. It highlighted that when discussing ‘teachers’, personalisation refers to targeted advertising and talent analytics, not just teaching and learning tools used in the classroom. It also materialised these conceptual unpacking by aligning to *The Report*’s simulations and scenarios.

This conceptual paper has provided a distinctive and much-needed viewpoint to add to the dominant discourse about the notion of innovative technology that uses or provides personalisation in educational settings. Drawing on *The Report’s* discussion of automated decision making and algorithmic bias but positioned in the consumer context of Australian K-12 teachers and technology in education, the paper unpacked practical implications for teachers who consume educational technology in their workplace. Data, analytics and AI are interconnected, and profitable components of contemporary educational settings and their consequences require policy enacted to protect teachers’ workplaces.

## Conclusion

The paper concludes by calling for empirical research into the size and scope of how teachers are learning about algorithmic bias either through teacher training programmes or professional development to enhance this discourse further. Digital teaching and learning tools produce intangible consequences for teachers’ workplace conditions. When considered as consequences according to recommendations made by the Australian Human Rights Commission, we can see a much more complex notion than what may be currently part of the current discourse.

To that end, how and to what extent the algorithmic systems used by commercial platforms as part of educational practice are modulating the classroom and shaping teachers’ working conditions requires a more significant discussion. There is a need to elaborate on rights-based discourse concerning AI for further consideration. Although this paper affords the reader interesting concepts to explore in terms of how the individual teacher and the collective (teachers) are situated in rights-based discourse and anti-discrimination specifically; the tensions discussed in this paper between individuals’ interest in engaging with technology and the broader implications of its use for the individual teacher and collective (teachers) require further consideration. Discussion, not only within schools and teachers but also by those who design policy and consider legislation associated with workplace and human rights. By doing so, further research can collectively grow towards effective partnerships and understand where the broader contexts are embedded and have an impact.
